# Effects of Homeopathic Arsenicum Album, Nosode, and Gibberellic Acid Preparations on the Growth Rate of Arsenic-Impaired Duckweed (*Lemna gibba* L.)

**DOI:** 10.1100/tsw.2010.202

**Published:** 2010-11-04

**Authors:** Tim Jäger, Claudia Scherr, Meinhard Simon, Peter Heusser, Stephan Baumgartner

**Affiliations:** ^1^ Institute of Complementary Medicine KIKOM, University of Bern, Switzerland; ^2^ Research Institute of Organic Agriculture, Frick, Switzerland; ^3^ Society for Cancer Research Hiscia Institute, Arlesheim, Switzerland; ^4^ Institute for Chemistry and Biology of the Marine Environment, University of Oldenburg, Germany; ^5^ Center for Integrative Medicine, University of Witten/Herdecke, Germany

**Keywords:** Lemna gibba, duckweed, homeopathy, arsenic, Arsenicum album, nosode, gibberellic acid

## Abstract

This study evaluated the effects of homeopathically potentized Arsenicum album, nosode, and gibberellic acid in a bioassay with arsenic-stressed duckweed (*Lemna gibba* L.). The test substances were applied in nine potency levels (17x, 18x, 21x–24x, 28x, 30x, 33x) and compared with controls (unsuccussed and succussed water) regarding their influence on the plant’s growth rate. Duckweed was stressed with arsenic(V) for 48 h. Afterwards, plants grew in either potentized substances or water controls for 6 days. Growth rates of frond (leaf) area and frond number were determined with a computerized image analysis system for different time intervals (days 0–2, 2–6, 0–6). Five independent experiments were evaluated for each test substance. Additionally, five water control experiments were analyzed to investigate the stability of the experimental setup (systematic negative control experiments). All experiments were randomized and blinded. The test system exhibited a low coefficient of variation (≈1%). Unsuccussed and succussed water did not result in any significant differences in duckweed growth rate. Data from the control and treatment groups were pooled to increase statistical power. Growth rates for days 0–2 were not influenced by any homeopathic preparation. Growth rates for days 2–6 increased after application of potentized Arsenicum album regarding both frond area (*p* < 0.001) and frond number (*p* < 0.001), and by application of potentized nosode (frond area growth rate only, *p* < 0.01). Potencies of gibberellic acid did not influence duckweed growth rate. The systematic negative control experiments did not yield any significant effects. Thus, false-positive results can be excluded with high certainty. To conclude, the test system with *L. gibba* impaired by arsenic(V) was stable and reliable. It yielded evidence for specific effects of homeopathic Arsenicum album preparations and it will provide a valuable tool for future experiments that aim at revealing the mode of action of homeopathic preparations. It may also be useful to investigate the influence of external factors (e.g., heat, electromagnetic radiation) on the effects of homeopathic preparations.

### INTRODUCTION

Specific homeopathic remedy effects are still the subject of controversy. Quantitative meta-analyses of randomized clinical trials covering all kinds of indications yielded inconclusive evidence for the efficacy of homeopathic remedies and seemed to be dependent on the inclusion criteria applied[1,2,3,4,5]. When restricted to specific medical conditions, quantitative meta-analyses of randomized controlled trials in the majority of cases reported significant homeopathic remedy effects compared to placebo[6,7,8,9,10,11]. Thus, it seems that – at least in certain cases – the dilution medium may adopt specific properties related to the mother tincture potentized, even without any molecules of the latter being present. However, no theoretical model exists at present that explains the mode of action of these highly diluted remedies according to the current scientific paradigm. Furthermore, reproducibility of results represents an ongoing challenge[12].

Based on the assumption that a characteristic feature of homeopathic preparations is to induce equilibrating effects, test systems with impaired organisms are expected to yield a more pronounced effect after application of homeopathic preparations compared to test systems using healthy organisms. However, stressing the organisms with external noxae to induce impairment usually leads to a considerable increase in variance[13]. Hence, it is very important to achieve a high degree of standardization and a standard deviation that is as low as possible.

We recently developed a new experimental method for homeopathic basic research that utilizes impaired organisms[14]. We used duckweed (*Lemna gibba* L.), a water plant that has often been employed as a research organism in standardized bioassays in ecotoxicology[15,16,17]. Furthermore, unimpaired (healthy) duckweed has recently been introduced in homeopathic basic research[18,19]. In an experimental preselection, arsenic(V) was chosen as the stressor because of its small variance. Arsenic has also repeatedly been investigated in ecotoxicological studies with duckweed[20,21,22,23]. Regarding arsenic concentration, a dose had to be found that, on the one hand, enabled a good measurable toxic effect and, on the other hand, permitted a vitality level ensuring sufficient self-healing power of the organisms. In a certain range, the arsenic concentration and, consequently, the degree of plant damage are positively correlated with standard deviation. An even further increase of the arsenic concentration leads to a decrease in standard deviation since the plants eventually die. Hence, the test system had to be stabilized (e.g., by establishing a homogeneous light field in the growth chamber and by careful selection of duckweed plants after the arsenic stress period) without losing the sensitivity of the system towards homeopathic treatment. Subsequently, we screened several test substances in homeopathic formulations regarding their capacity to alleviate the stress induced[14]. In these investigations, homeopathic Arsenicum album and nosode preparations increased the growth rate of duckweed consistently over two evaluation approaches, and thus seemingly reduced the stress induced by arsenic(V).

The aim of the present study was to investigate whether the effects of Arsenicum album and nosode preparations could be confirmed in further independent reproduction experiments. We additionally included gibberellic acid as a homeopathic test substance in the present experimental series with arsenic(V)-stressed duckweed. This was done in order to compare the results to former experiments on healthy duckweed, where specific effects of homeopathically potentized gibberellic acid had been observed[19]. Arsenicum album, nosode, and gibberellic acid were applied in nine potency levels (17x, 18x, 21x–24x, 28x, 30x, 33x) and compared with controls (unsuccussed and succussed water) regarding their influence on the plant's growth rate. The final evaluation included five independent experiments for each test substance. To control test system stability, five independent systematic negative control experiments were conducted during the entire time span of the investigations. All experiments were coded (blinded) and applied in randomized order to avoid experimental biases.

## MATERIALS AND METHODS

### General Experimental Design

A single experiment comprised 100 beakers with *Lemna gibba* (Fig. 1). For every experimental parameter (n = 20 in total, n = 18 letter-coded samples and two open control conditions, see below), five replicates were used and randomly allocated in a fixed-blocked randomization scheme. The 18 coded samples consisted either of nine potency levels (17x, 18x, 21x–24x, 28x, 30x, 33x) of a given substance and of nine independent control preparations (four samples unsuccussed water and five samples one-time succussed water), or – in the case of the systematic negative control experiments – of 18 unsuccussed water samples coming from the same source. After preparation, all test solutions were randomized and coded (blinded) by a person not involved in the experiments. Duckweed was stressed with arsenic(V) for 48 h. Subsequently, the plants grew in either potentized substances or water controls for 6 days. Growth rate and color of fronds were determined for different time intervals (days 0–2, 2–6, 0–6).

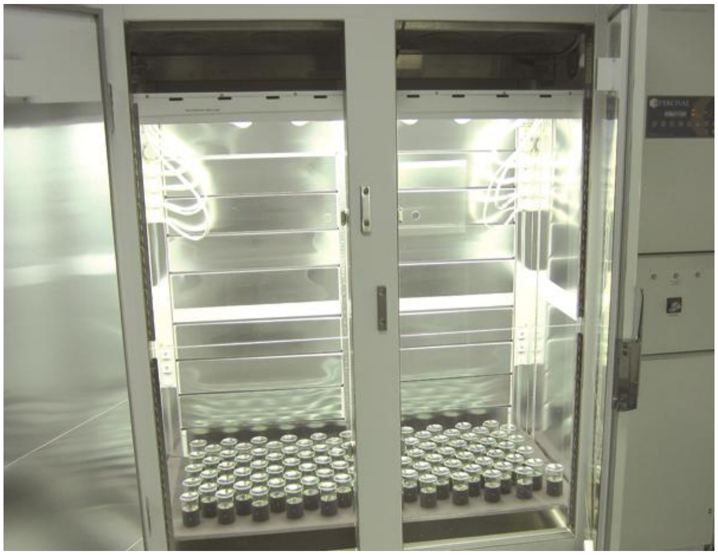

**FIGURE 1**. Experimental setup of a single experiment in the growth chamber (100 beakers with *L. gibba*). For every experimental parameter (n = 20 in total), five replicates were used and allocated in a fixed-blocked randomization scheme. The 20 experimental conditions consisted of 18 letter-coded samples and two additional open controls, one with unimpaired duckweed and one with duckweed impaired during the entire experimental interval (the latter two controls were not used for the statistical evaluation).

In a screening, a total of 12 experiments had been performed with arsenic-impaired duckweed, with 11 different potentized substances and one systematic negative control experiment[14]. Out of the 11 substances tested, we selected Arsenicum album, nosode, and gibberellic acid, and performed four additional independent experiments for each substance, designed as identical repetitions of the initial screening experiment (see Fig. 2). Furthermore, we conducted four additional full-size experiments with pure water as the only treatment parameter (systematic negative control experiments) to investigate the stability of the experimental setup over the entire study period. Thus, a total of 16 new experiments were conducted between April and September of 2009. For the statistical evaluation, data from the screening experiments[14] were pooled with those of the newly performed experiments. Thus, a total of 20 experiments (four experimental series with five independent experiments each) entered the final dataset for evaluation.

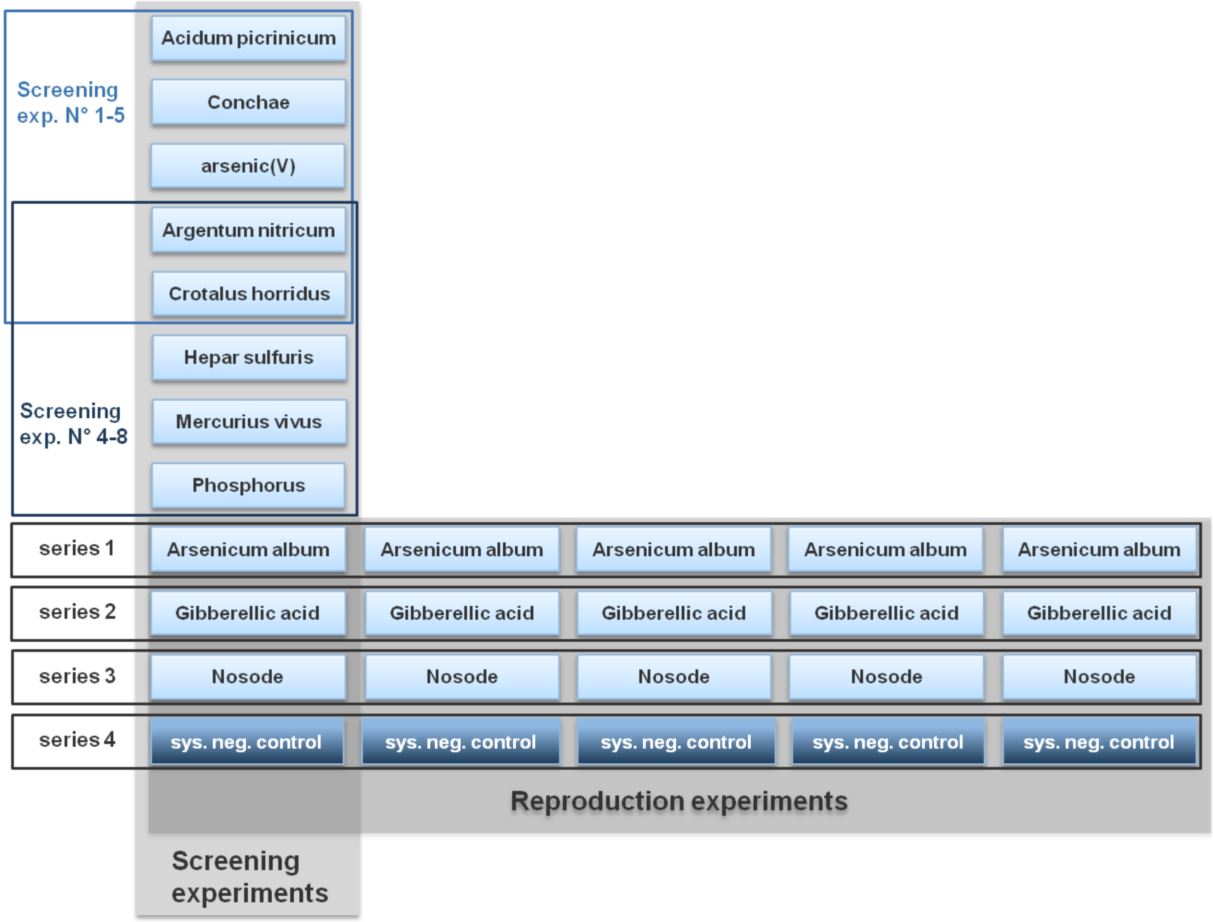

**FIGURE 2.** Diagram of all analyzed experiments. One box corresponds to one single experiment. The present data evaluation comprised for each of the experimental series (series 1–4) five independent experiments (sys. neg. control = systematic negative control experiment). Additional control calculations were made with two groups of eight screening experiments (N° 1–5 and N° 4–8). Data of the screening experiments were evaluated and published elsewhere[14].

### Preparation of Potentized Test Solutions and Controls

A detailed description of the sample preparation has been given in a precursor publication[14]. Briefly, all test solutions for one experiment (potencies and controls) were prepared freshly, in accordance with the multiple glass method, between 6 and 9 a.m. on the day of the experiment from the same batch of distilled (Büchi, Fontavapor-250, Flawil, Switzerland) and autoclaved (Getinge AB-Typ-GE-406, Sweden) water.

For preparation of the nosode, duckweed grew for 48 h in 2000-ml moStM (see below) comprising 158 mg/l arsenic(V). Duckweed was cut into small pieces, put into 85 ml of distilled water and 15 ml of ethanol (94%, Alcosuisse-S15-sekunda, Schachen, Switzerland), and agitated for 2 h (Turbula T2 C, Willy A. Bachofen AG, Basel, Switzerland) in an Erlenmeyer flask of Duran® glass (250 ml, Schott, Mainz, Germany). After maceration at 20°C under diffused light for 21 days, the extract was filtered (Macherey-Nagel, MN-619-eh ¼ Ø 185mm, Germany) and stored at 4°C for 12 days. Gibberellic acid (Sigma-Aldrich, Buchs, Switzerland) was potentized in acetone (AppliChem A2300 Darmstadt, Germany) to 1x, then further on in distilled water. Arsenicum album was obtained in the lowest potency available (5x, Weleda, Arlesheim, Switzerland). All samples were further potentized in distilled water.

For the potentization process, which was designed by the main experimenter (TJ), Erlenmeyer flasks of Duran® glass (≤6x: 250 ml, ≥7x: 500 ml, Schott, Mainz, Germany) were used. 15 ml of potency stock solution was added to 135 ml of distilled water. Then the Erlenmeyer flask was agitated once upside-down with a horizontal drive to generate a macroscopic laminar vortex. After calming of the vortex, the flask was struck straight down and stopped abruptly to induce a chaotic turbulent movement in water. These two steps – combining both a well-structured laminar vortex flow and a chaotic movement of water – were repeated ten times. For the next potency level, 15 ml of this solution were added to the next potentization vessel containing 135 ml of distilled water and agitated in the same manner. At potency level 7x, flask size was changed from 250 to 500 ml, and the filling volume rose to 350 ml; thus, 35 ml of the former potency level were added to 315 ml of distilled water. This process of successive tenfold dilution steps and vigorous shaking proceeded until the potency step 33x was accomplished.

Two types of controls were prepared: unsuccussed water (c0) and succussed water (c1), corresponding to water 1x, shaken analogously to the potencies described above. Four samples of unsuccussed water were prepared in four 500-ml Erlenmeyer flasks and five samples of succussed water in five analogous Erlenmeyer flasks. These controls were chosen according to the considerations discussed in detail elsewhere[24]. In short, comparison of unsuccussed and succussed controls allows the estimation of the influence of the unspecific physicochemical effects induced by agitation (e.g., increased ion dissolution, radical formation, pH changes due to $CO_2$ concentration changes, etc.) that might lead to false-positive conclusions regarding the specific efficacy of homeopathic dilutions. The combined use of unsuccussed and succussed controls yields more information than the use of potentized solvent alone.

From the potencies prepared, nine potency levels (17x, 18x, 21x–24x, 28x, 30x, 33x) were used for the experiments. Together with the nine control preparations (see above), 18 samples were prepared in total. These 18 test solutions were randomized and coded (blinded) by a person not involved in the experiments by manual random assignment of a double letter code from a predefined list.

### Experimental Procedure

For the *Lemna* bioassay, arsenic (pure) stock cultures of duckweed *L. gibba* L. (clone no. 9352) were grown (according to a standard of the International Organization for Standardization[17]) first on solid, then in liquid-modified Steinberg medium (moStM, all ingredients Fluka, Buchs, Switzerland) to acclimatize the plants to the experimental conditions and get large amounts of plants under controlled laboratory conditions. The medium was changed weekly to achieve rapid growth, close to exponential growth, and it was assured that growth would not be restricted (e.g., due to space limitations or nutrient restrictions).

The last change of moStM was 48 h before starting the experiment. Plants were transferred to one vessel containing 2000 ml of freshly prepared moStM to ensure identical nutrient concentration when adding 158 mg/l arsenic(V) ($AsHNa_2O_4 \times 7H_2O$, Sigma-Aldrich, Buchs, Switzerland). Fronds that were malformed or severely damaged (Fig. 3) were removed from the vessel 24 h before starting the experiment. After 48 h of intoxication, arsenic-treated duckweed exhibited an area-related growth rate ($r_{(area)}$) of approximately 44% compared to duckweed growing without arsenic ($r_{with\ arsenic} = 0.16\ d^{-1}$, $r_{without\ arsenic} = 0.36\ d^{-1}$).

On the day of the experiment, plants without visible lesions, chlorosis, or necrosis were selected from the vessel (≈1.5%). Test specimens were sorted according to number of fronds, similar size, color, and form. Then they were used as inoculum for all beakers containing test solutions or controls, respectively.

A single experiment comprised 100 beakers (Fig. 1). N = 20 experimental parameters were investigated in five replicate beakers each (20 × 5 = 100 beakers). The 20 parameters consisted of 18 letter-coded samples (nine potency levels of a given substance and nine control preparations, see above) and two additional open control conditions (parameters), one with unimpaired duckweed and one with duckweed impaired with arsenic(V) during the entire experimental interval. The latter two controls did not enter the statistical evaluation.

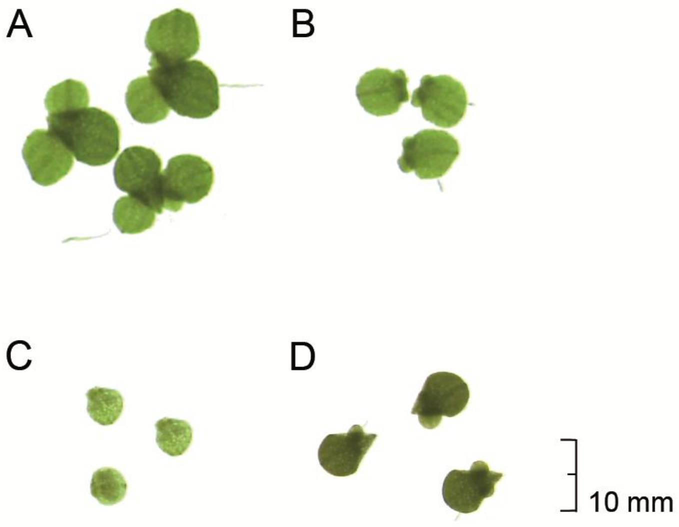

**FIGURE 3.** Duckweed (*L. gibba* L.) fronds (leaves): (A) unimpaired (healthy) fronds, (B) arsenic(V)-impaired fronds as used in the experiments. Too severely damaged fronds (C) or peak-shaped fronds (D) were not used in the experiments.

For each experiment, 50 ml of moStM was poured (Bottletop dispensing head, 50 ml, Wertheim, Germany) in 100 beakers each (150 ml, SIMAX®, Kavalier, Sázava, Czech Republic). Then 50 ml of 18 coded samples in five replicates each was added to 90 beakers. For the two open control conditions, 50 ml of distilled water were added to five beakers each, and 50 ml of aqueous arsenic(V) (158 mg/l) were added to another five beakers each.

The sorted impaired duckweed colonies were carefully put into the 90 beakers at random. Into the 10 beakers of the two open controls, sorted unimpaired duckweed was placed. Frond area and frond number per beaker were measured at the beginning of the experiment (day 0), and on days 2 and 6 using an image processing system (Scanalyzer, duckweed analytic software, version 4, LemnaTec, Aachen, Germany).

Experiments were conducted in a plant growth chamber (AR-75L, Percival Scientific, Boone, Iowa) illuminated with fluorescent lights (137 ± 0.6 μmol photons $m^{-2}$ $sec^{-1}$ PAR, F32 T8/TL 741, Philips, U.S.) for 24 h. Mean air temperature was 21.5 ± 0.5°C, mean temperature of moStM was 22.4 ± 0.3°C (Endotherm, Dornach, Switzerland), and mean relative humidity was 68 ± 5% (Ebro EBI-20-TH, Ingolstadt, Germany).

From the measured frond area and frond number, the average growth rate per day ($r_{(area)}$, $r_{(number)}$) was calculated for three time intervals (days 0–2, 2–6, and 0–6) according to the equation: $r = (\ln x_{t2} - \ln x_{t1}) / (t_2 - t_1)$ where $x_{t1}$ is the value of observation parameter at day $t_1$, $x_{t2}$ is the value of observation parameter at day $t_2$, and $t_2 - t_1$ is the time interval between $x_{t1}$ and $x_{t2}$ in days. More details concerning the methodological procedures of the *Lemna* bioassay were described elsewhere[14].

### Statistical Analysis

All experiments (four screening experiments[14] and four reproduction series with four experiments each) yielded a total of 10,800 data points (20 experiments × 90 beakers × 3 time points × 2 observation parameters) that were transformed into 10,800 growth rate data values for the final statistical evaluation. The data from eight measurements are missing due to software failures and spilling of beakers. All other data were included into the statistical analysis.

Data from the five systematic negative control experiments were used to estimate the variability of the bioassay. We grouped the data of the 90 beakers of every single experiment into 18 groups of five replicates (beakers) and calculated mean values for these 18 subgroups for frond area– and front number–related specific growth rate (days 0–2, 2–6, 0–6 each). Based on these 18 values, the coefficient of variation (CV) was calculated for every single experiment and time interval.

Regarding a possible succussion effect, data of the unsuccussed (c0) and successed (c1) water controls of experiments with potentized substances were analyzed using a two-way analysis of variance (ANOVA) *F*-test for independent samples. Data from the water control experiments were not used since the systematic negative control experiments included only unsuccussed water.

A comparison of growth rate data ($r_{(area)}$ and $r_{(number)}$) between pooled potencies and pooled water controls (succussed and unsuccussed) was evaluated for statistical significance based on two-way ANOVA *F*-tests for independent samples. Data of every experiment were normalized to the control groups. In all statistical analyses, the level of significance was $\alpha = 0.05$. An interaction term between experiment number and treatment was included in the statistical model in order to be able to observe possible effect-modulating factors associated with the date of the experiment. Planned comparisons were evaluated with the LSD test only if the corresponding global *F*-test was significant ($p < 0.05$) (protected Fisher's LSD). This constitutes a good safeguard against type I as well as type II errors[25].

Levene's test was conducted to determine homogeneity of variances. Normal data distribution and skewness was evaluated graphically by quantile-quantile plots. No evident deviations from normality were observed. Due to the central limit theorem and the large amount of data in our study, slight deviations from normality are irrelevant. Furthermore, appropriateness of the statistical evaluation was checked by the evaluation of the systematic negative control experiments. All data were analyzed using the software STATISTICA Version 6 (Stat Soft, Tulsa, OK).

### RESULTS AND DISCUSSION

#### Control Experiments

The stability of the experimental setup was investigated in five systematic negative control experiments. These revealed very small coefficients of variation for all outcome parameters measured (≈1%, cf. Table 1). In this respect, the bioassay with impaired duckweed is superior to other model systems with impaired plants used in homeopathic basic research, since typical coefficients of variation are in the order of 10–80%[26,27,28]. Hence, we conclude that our newly developed test system with arsenic-impaired duckweed showed a very low standard deviation.

In the statistical analysis (performed in an absolutely identical manner as in the experiments with potentized substances, see below) the global ANOVA *F*-tests yielded no significant effects for any outcome parameter calculated, neither for treatment (here 18 "pseudo-treatments", distilled water only) nor for the interaction of treatment with experiment number (Table 2, Series SNC). Thus, false-positive results caused by uncontrolled influences during the experiment (e.g., systematic errors due to spatial gradients in light intensity or temperature) could be excluded with very high certainty (see also section below, Additional Control Calculations).

#### Succussion Effect

In order to account for unspecific physicochemical effects occurring during the succussion step of the potentization process (e.g., increased ion dissolution from the vessel walls, pH alteration due to $CO_2$ dissolution, etc.), unsuccussed and succussed water controls from all experiments with potentized substances were compared, as proposed by Baumgartner et al.[24]. In ANOVA *F*-tests of growth rate data, no significant succussion effect and, with one exception, no significant interaction with experiment

**TABLE 1**
**Coefficient of Variation (CV)* for Each Outcome Parameter in the Five Systematic Negative Control Experiments (SNC)**

| Experiment N° | Growth Rate $r_{(area)}$ | | | Growth Rate $r_{(number)}$ | | |
|---|---|---|---|---|---|---|
| | Day 0–2 | Day 2–6 | Day 0–6 | Day 0–2 | Day 2–6 | Day 0–6 |
| SNC Exp. N° 1 | 1.57 | 0.91 | 1.05 | 1.24 | 1.01 | 0.84 |
| SNC Exp. N° 2 | 0.93 | 0.72 | 0.72 | 1.09 | 1.25 | 0.58 |
| SNC Exp. N° 3 | 1.04 | 0.69 | 0.81 | 0.81 | 0.87 | 0.66 |
| SNC Exp. N° 4 | 0.94 | 0.95 | 0.69 | 0.42 | 0.88 | 0.63 |
| SNC Exp. N° 5 | 0.65 | 0.5 | 0.63 | 0.9 | 0.89 | 0.67 |
| Mean | 1.03 | 0.76 | 0.78 | 0.89 | 0.98 | 0.68 |

* CV was calculated based on mean values of 18 groups of five replicates (total 90 beakers) in one experiment.

**TABLE 2**
**ANOVA Analysis of the Four Main Experimental Series**

| Experimental Series | Effects | *p* Values for Growth Rate $r_{(area)}$ | | | *p* Values for Growth Rate $r_{(number)}$ | | |
|---|---|---|---|---|---|---|---|
| | | Day 0–2 | Day 2–6 | Day 0–6 | Day 0–2 | Day 2–6 | Day 0–6 |
| Arsenicum album | 1: Exp. No. | 0.746 | 0.773 | 0.745 | 0.835 | 0.505 | 0.34 |
| | 2: Treatment | 0.098 | **<0.001** | **<0.001** | 0.143 | **<0.001** | **0.001** |
| | 1/2: Interaction | 0.746 | 0.773 | 0.745 | 0.835 | 0.505 | 0.34 |
| Nosode | 1: Exp. No. | 0.893 | 0.237 | 0.649 | 0.372 | 0.238 | 0.113 |
| | 2: Treatment | 0.971 | **0.008** | 0.103 | 0.418 | 0.073 | **0.036** |
| | 1/2: Interaction | 0.893 | 0.237 | 0.649 | 0.372 | 0.238 | 0.113 |
| Gibberellic acid | 1: Exp. No. | 0.929 | 0.726 | 0.739 | 0.974 | 0.292 | 0.353 |
| | 2: Treatment | 0.565 | 0.992 | 0.772 | 0.988 | 0.661 | 0.765 |
| | 1/2: Interaction | 0.929 | 0.726 | 0.739 | 0.974 | 0.292 | 0.353 |
| SNC | 1: Exp. No. | 0.728 | 0.639 | 0.649 | 0.869 | 0.899 | 0.958 |
| | 2: Treatment | 0.72 | 0.961 | 0.89 | 0.751 | 0.288 | 0.374 |
| | 1/2: Interaction | 0.728 | 0.639 | 0.649 | 0.869 | 0.899 | 0.958 |

*Note:* Test substances Arsenicum album, nosode, and gibberellic acid, as well as systematic negative control experiments (SNC) with the independent parameters experiment number (n = 5, independent experiments) and treatment (n = 2, potencies vs. controls). Data for the nine potency levels (17x, 18x, 21x–24x, 28x, 30x, 33x) and the nine control samples (four samples unsuccussed water, five samples succussed water) were pooled. Measurement parameters were frond area– and frond number–related growth rates for different time intervals (days 0–2, 2–6, 0–6). Data were normalized to the mean of the pooled water controls for every individual experiment. Significant values ($p < 0.05$) are shown in bold.

number were observed for any outcome parameter (Table 3). Since succussed water (c1) essentially did not differ from unsuccussed water (c0) in its effects on duckweed growth rate, we concluded that possible unspecific effects due to the succussion procedure were negligible in this test system. Therefore, effects of potentized substances (see below) were compared to the pooled data from both control groups (defined as control c) in order to increase statistical power, and to balance the number of samples in the group with pooled potencies and the group of controls.

**TABLE 3**
**Comparison (ANOVA *F*-Tests) of Unsuccussed (c0) and Successed (c1) Controls by Two Growth Parameters in Three Time Intervals (Days 0–2, 2–6, 0–6)**

| Experimental Series | Effects | *p* Values for Growth Rate $r_{(area)}$ | | | *p* Values for Growth Rate $r_{(number)}$ | | |
|---|---|---|---|---|---|---|---|
| | | Day 0–2 | Day 2–6 | Day 0–6 | Day 0–2 | Day 2–6 | Day 0–6 |
| Arsenicum album | 1: Exp. No. | 1.000 | 1.000 | 1.000 | 1.000 | 1.000 | 1.000 |
| | 2: Treatment | 0.583 | 0.356 | 0.383 | 0.171 | 0.633 | 0.458 |
| | 1/2: Interaction | 0.994 | 0.518 | 0.789 | 0.510 | 0.096 | 0.222 |
| Nosode | 1: Exp. No. | 1.000 | 1.000 | 1.000 | 1.000 | 1.000 | 1.000 |
| | 2: Treatment | 0.919 | 0.325 | 0.579 | 0.072 | 0.070 | 0.456 |
| | 1/2: Interaction | 0.260 | 0.754 | 0.523 | 0.069 | 0.226 | 0.800 |
| Gibberellic acid | 1: Exp. No. | 1.000 | 1.000 | 1.000 | 1.000 | 1.000 | 1.000 |
| | 2: Treatment | 0.632 | 0.225 | 0.278 | 0.755 | 0.213 | 0.180 |
| | 1/2: Interaction | 0.886 | 0.645 | 0.626 | **0.026** | 0.771 | 0.616 |

*Note:* Effects were calculated for three experimental series (test substances Arsenicum album, nosode and gibberellic acid) with five independent experiments each. Data were normalized to the mean of the pooled water controls for every individual experiment. Significant values ($p < 0.05$) are shown in bold.

### Experiments with Potentized Substances: Global Effects

Duckweed growth rate data (area- and number-related growth rates for the three time intervals) for the three main experimental series (treatment with Arsenicum album, nosode, and gibberellic acid) were analyzed separately, always in full two-way ANOVA with the independent variables treatment (n = 2, all potency levels vs. both controls) and experiment number (1–5). Results are given in Table 2 (series Arsenicum album, nosode, and gibberellic acid) and in Fig. 4 for the area-related growth rate (days 2–6).

There were differences in absolute growth rates varying from experiment to experiment (cf., Fig. 4). Experiments of a given test substance were not conducted one after the other. Systematic negative control experiments and experiments with test substances were conducted in randomized order. Therefore, the seemingly decreasing trend in absolute growth rates of the nosode experiments (Fig. 4B) has no specific meaning. We estimated the coefficient of variation for the absolute values of growth rate $r_{(area)}$ (days 2–6) over the entire experimental period on the basis of the pool of control data (c0, c1) from all experiments with homeopathic potencies. It averages to 3.1% (mean $0.42 \pm 0.02\ d^{-1}$).

Homeopathic potencies of Arsenicum album and nosode enhanced the growth rate of impaired *L. gibba*. Application of potentized Arsenicum album yielded the largest effects compared to water controls for the outcome parameters frond area (growth rate $r_{(area)}$ days 2–6: $p < 0.001$, and days 0–6: $p < 0.001$) and frond number (growth rate $r_{(number)}$ days 2–6: $p < 0.001$, and days 0–6: $p < 0.001$, Table 2). In all five single experiments with Arsenicum album, growth rates of samples with potencies numerically exceeded those of controls (Fig. 4A). Application of potentized nosode preparations also yielded significant effects on duckweed's frond area and frond number (growth rate $r_{(area)}$ for days 2–6: $p < 0.01$; growth rate $r_{(number)}$ for days 0–6: $p = 0.036$, Table 2), but only in four experiments did growth rates of duckweed treated with potencies numerically exceed those of the control plants (Fig. 4B). Since the interaction between treatment and experiment number was not significant, the effects of potentized Arsenicum album and nosode seemed to be reproducible (within the limits of statistical power). Potencies of gibberellic acid did not exert any significant effects (Table 2, Fig. 4C). Growth rates in the first time interval (days 0–2) were not influenced by any homeopathic treatment. The systematic negative control experiments did not yield any evidence for systematic errors associated with the experimental setup (Table 2, Fig. 4D).

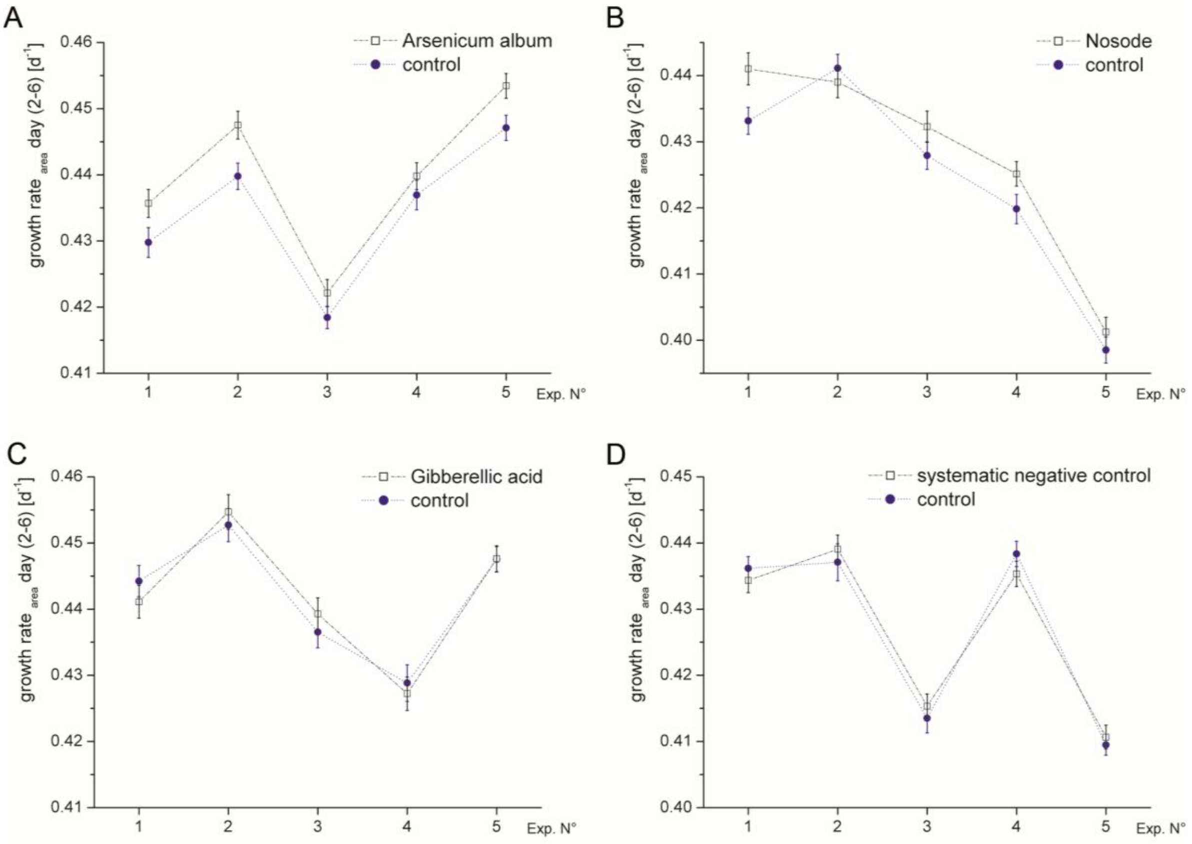

**FIGURE 4.** Growth rate of *L. gibba* L. ($r_{(area)}$ days 2–6) [$d^{-1}$] (mean ± standard error) treated with different homeopathic preparations: (A) Arsenicum album; (B) nosode; (C) gibberellic acid. Data for the nine potency levels (17x, 18x, 21x–24x, 28x, 30x, 33x) were pooled and compared to the pooled data for the nine control samples (four samples unsuccussed water, five samples succussed water). The systematic negative control experiment (D) compared 45 randomly allocated samples of unsuccussed water with another 45 randomly allocated samples of unsuccussed water. All four experimental series (A–D) comprised five independently performed experiments (Exp. N°). Lines connecting data points are no interpolations.

### Experiments with Potentized Substances: Effects of Single Potency Levels

Duckweed growth rate data (area- and number-related growth rates for the three time intervals) were normalized to the pooled control data set. The three main experimental series (treatment with Arsenicum album, nosode, and gibberellic acid) were analyzed separately, always in full two-way ANOVA with the independent variables treatment (n = 11, nine potency levels and two controls) and experiment number (1–5). Results are given in Table 4 (series Arsenicum album, nosode, and gibberellic acid) and in Fig. 5 for area-related growth rate (days 2–6). The systematic negative control experiments were analyzed analogously, with randomized allocation of the beakers to pseudo-treatment parameters (w0–w10).

In this analysis, significant homeopathic treatment effects were observed for the Arsenicum album series only, and were most pronounced for the area related growth rate for days 2–6. Regarding single potency levels, 18x, 21x, 22x, 23x, and 33x of Arsenicum album significantly enhanced the main outcome parameter growth $rate_{(area)}$ days 2–6. None of the single potency levels (17x–33x) decreased the growth rate.

No single potency levels of nosode significantly enhanced the growth rate, in contrast to the analysis of the pooled data (see above). Numerically, however, all nosode potency levels exhibited a larger growth rate than both controls (Fig. 5). The effect of the nosode treatment seemed to be weaker than the Arsenicum album treatment, leading to significant effects only after pooling data from all potency levels.

**TABLE 4**
**ANOVA Analysis of the Four Main Experimental Series Regarding Single Potency Levels**

| Experimental Series | Effects | *p* Values for Growth Rate $r_{(area)}$ | | | *p* Values for Growth Rate $r_{(number)}$ | | |
|---|---|---|---|---|---|---|---|
| | | Day 0–2 | Day 2–6 | Day 0–6 | Day 0–2 | Day 2–6 | Day 0–6 |
| Arsenicum album | 1: Exp. No. | 0.453 | 0.507 | 0.462 | 0.637 | 0.200 | 0.083 |
| | 2: Treatment | 0.179 | **0.007** | **0.023** | 0.466 | 0.074 | 0.068 |
| | 1/2: Interaction | 0.872 | 0.921 | 0.967 | 0.948 | 0.829 | 0.915 |
| Nosode | 1: Exp. No. | 0.713 | **0.039** | 0.325 | 0.094 | **0.032** | **0.008** |
| | 2: Treatment | 0.906 | 0.520 | 0.769 | 0.122 | 0.171 | 0.284 |
| | 1/2: Interaction | 0.207 | 0.976 | 0.830 | 0.224 | 0.823 | 0.845 |
| Gibberellic acid | 1: Exp. No. | 0.796 | 0.380 | 0.453 | 0.935 | 0.058 | 0.092 |
| | 2: Treatment | 0.935 | 0.785 | 0.862 | 0.894 | 0.605 | 0.854 |
| | 1/2: Interaction | 0.868 | 0.674 | 0.836 | 0.346 | 0.771 | 0.905 |
| SNC | 1: Exp. No. | 0.437 | 0.300 | 0.337 | 0.633 | 0.697 | 0.883 |
| | 2: Treatment | 0.309 | 0.641 | 0.521 | 0.805 | 0.366 | 0.957 |
| | 1/2: Interaction | 0.952 | 0.979 | 0.999 | 0.349 | 0.178 | 0.968 |

*Note:* Test substances Arsenicum album, nosode, and gibberellic acid, as well as systematic negative control experiments (SNC) with the independent parameters experiment number (n = 5, independent experiments) and treatment (n = 11, nine potency levels [17x, 18x, 21x–24x, 28x, 30x, 33x] and two controls [c0, c1]). Measurement parameters were frond area– and frond number–related growth rates for different time intervals (days 0–2, 2–6, 0–6). Data were normalized to the mean of the pooled water controls for every individual experiment. Significant values ($p < 0.05$) are shown in bold.

### Additional Control Calculations

We performed several control calculations to ensure the validity of the study results: (1) a sensitivity analysis of the Arsenicum album growth rate stimulation effects; (2) an allocation of the water control experiments according to the randomization schemes of the Arsenicum album, nosode, and gibberellic acid experimental series; and (3) a further analysis of the screening experiments[14].

1. We performed a sensitivity analysis regarding the stability of the growth stimulation effects induced by Arsenicum album onto the growth rate $r_{(area)}$ days 2–6. This analysis is based on the two-way ANOVA with the independent variables treatment (n = 2, all potency levels vs. both controls) and experiment number (1–5). Eliminating growth rate extreme values ("outliers") in a wide range (3.25 × SD – 1.75 × SD) essentially did not influence the significance levels of the *F*-test for the main ANOVA treatment effect (comparing either Arsenicum album with the pooled controls or comparing controls with controls in the systematic negative control experiment [Table 5]). Hence, the results are stable and not due to some extreme values.
2. The primary evaluation of the systematic negative control experiments was based on randomized allocations of the 18 × 5 beakers to the nine pseudo-treatment or nine pseudo-control groups per experiment (as for the experiments with homeopathic preparations, a person not involved in the experiments established five independent randomization lists for the five negative control experiments). One might argue that the randomized allocations, which were established for the verum experiments (with Arsenicum album, nosode, and gibberellic acid) might have generated false-positive results by chance (e.g., due to unidentified light or heat gradients in the growth chamber). To test this hypothesis, we analyzed the data from the five systematic negative control experiments with the randomization lists from the verum experiments (with Arsenicum album,

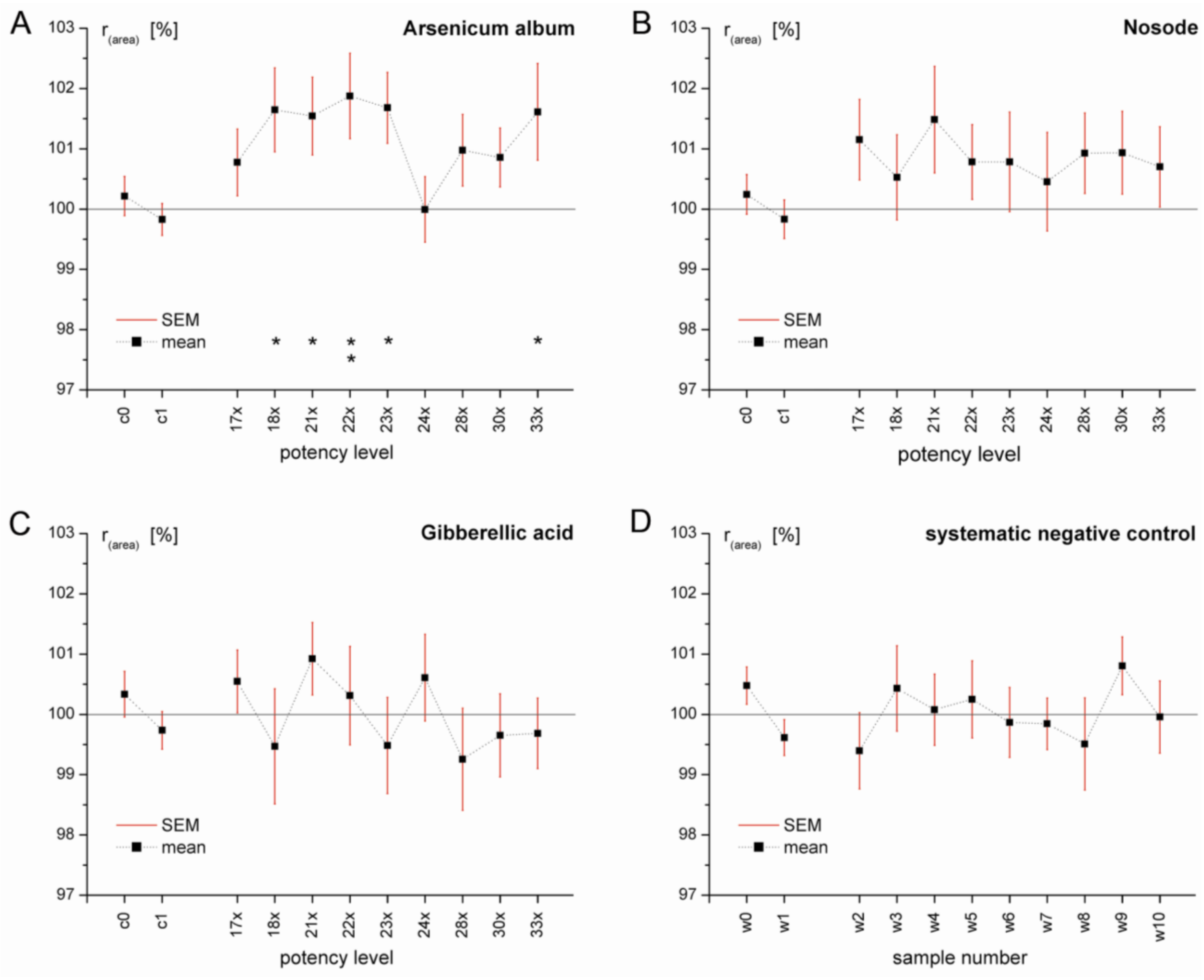

**FIGURE 5.** Area-related specific growth rates ($r_{(area)}$ days 2–6) [%] of *L. gibba* growing in different potency levels of selected test substances (A–C) in comparison to the corresponding water controls (c0 + c1). Part (D) shows the corresponding graph for the pure water control experiments (systematic negative controls) with samples of identical origin (unsuccussed water = dilution medium used). Mean values (dots) ± standard error (bars) for five independent experiments, respectively. Every data point for single potency levels is an average from five independent experiments with five replicates (beakers) each (n = 25 per data point plotted). The two data points for controls are an average from five independent experiments with 25 beakers (succussed controls) or 20 beakers (unsuccussed controls) (n = 125 and n = 100 per data point plotted). Data were normalized to the experimental mean of succussed and unsuccussed water controls (c0 + c1) for every individual experiment. Lines connecting data points are no interpolations. Statistically significant differences (Fisher's LSD test) between single potency levels and the pooled water control c are indicated by *($0.01 < p < 0.05$), **($p < 0.01$).

nosode, and gibberellic acid). The results of these ANOVA analyses did not yield any evidence for false-positive results due to the specific randomization lists used for the verum experiments (Table 6).

3. The experimenter was not blinded regarding the knowledge on whether a verum experiment (with Arsenicum album, nosode, and gibberellic acid) or a systematic negative control experiment had actually been carried out. Even though the experimenter was blinded regarding the potency or control treatment groups, one might argue that he might have influenced the experiment in a very subtle way, e.g., by working more carefully when carrying out a systematic negative control experiment or some other minor differences in experimental handling. The negative results of the gibberellic acid experimental series are, however, not in favor of this hypothesis. Additionally, we performed a control analysis of the screening experiments[14] without Arsenicum album, nosode,

**TABLE 5**
**ANOVA *F*-Test Statistics for the Main Treatment Effect of the Outcome Parameter Growth Rate $r_{(area)}$ Days 2–6 as a Function of Excluded Extreme Values (Limits 3.25 × SD – 1.75 × SD)**

| Experimental Series | Outlier Limit (x * SD) | 3.25 | 3 | 2.75 | 2.5 | 2.25 | 2 | 1.75 |
|---|---|---|---|---|---|---|---|---|
| Arsenicum album | Outliers [n] | 0 | 0 | 0 | 3 | 9 | 18 | 37 |
| | Outliers [%] | 0.0 | 0.0 | 0.0 | 0.7 | 2.0 | 4.0 | 8.2 |
| | *p* Value (potencies vs. controls) | 0.00004 | 0.00004 | 0.00004 | 0.00002 | 0.00007 | 0.00000 | 0.00000 |
| SNC | Outliers [n] | 0 | 1 | 3 | 6 | 9 | 14 | 30 |
| | Outliers [%] | 0.0 | 0.2 | 0.7 | 1.3 | 2.0 | 3.1 | 6.7 |
| | *p* Value (contols vs. controls) | 0.27024 | 0.32771 | 0.47507 | 0.74118 | 0.51314 | 0.44148 | 0.54617 |

*Note:* Treatment effect compared pooled data from Arsenicum album potency levels (17x, 18x, 21x–24x, 28x, 30x, 33x) with pooled data from both water controls (succussed and unsuccussed), or unsuccussed controls with unsuccussed controls in the systematic negative control experiments (SNC). Five independent experiments with test substance Arsenicum album or five independent negative controls experiments were included (SNC: 450 data points, Arsenicum album 448 data points in total).

**TABLE 6**
**ANOVA Analysis of the Systematic Negative Control Experiments (SNC) with the Randomization Lists of the Verum Experimental Series (Arsenicum album, Nosode, and Gibberellic Acid)**

| Randomization List | Statistical Parameters | *p* Values for Growth Rate $r_{(area)}$ | | | *p* Values for Growth Rate $r_{(number)}$ | | |
|---|---|---|---|---|---|---|---|
| | | Day 0–2 | Day 2–6 | Day 0–6 | Day 0–2 | Day 2–6 | Day 0–6 |
| Arsenicum album series | 1: Exp. No. | 0.526 | 0.341 | 0.471 | 0.885 | 0.636 | 0.730 |
| | 2: Treatment | 0.759 | 0.269 | 0.593 | 0.638 | 0.412 | 0.202 |
| | 1/2: Interaction | 0.526 | 0.341 | 0.471 | 0.885 | 0.636 | 0.730 |
| Nosode series | 1: Exp. No. | 0.943 | 0.495 | 0.825 | 0.624 | 0.700 | 0.273 |
| | 2: Treatment | 0.958 | 0.275 | 0.479 | 0.681 | 0.465 | 0.453 |
| | 1/2: Interaction | 0.943 | 0.495 | 0.825 | 0.624 | 0.700 | 0.273 |
| Gibberellic acid series | 1: Exp. No. | 0.255 | 0.552 | 0.962 | 0.728 | 0.608 | 0.773 |
| | 2: Treatment | 0.305 | 0.482 | 0.295 | 0.338 | 0.053 | 0.181 |
| | 1/2: Interaction | 0.255 | 0.552 | 0.962 | 0.728 | 0.608 | 0.773 |

*Note:* Independent parameters were experiment number (n = 5, independent experiments) and treatment (n = 2, 45 unsuccussed controls vs. 45 unsuccussed controls). Measurement (outcome) parameters were frond area– and frond number–related growth rates for different time intervals (days 0–2, 2–6, 0–6). Data were normalized to the mean of 45 pooled water controls for every individual experiment.

and gibberellic acid. The remaining eight screening experiments were allocated to two series of five single experiments each (Group 1: Exp. N° 1–5, arsenic(V), Hepar sulfuris, Mercurius vivus naturalis, Phosphorus, Conchae ; Group 2: Exp. N° 4–8, Phosphorus, Conchae, Acidum picrinicum, Argentum nitricum, Crotalus horridus; see Fig. 2) and statistically analyzed in exactly the same way as in the series with a repeatedly tested homeopathic substance (e.g., Arsenicum album). Also in these two analyses, no significant effects were observed (Table 7). We therefore conclude that it is very improbable that the treatment effects observed in the experimental series with Arsenicum album or nosode are due to unidentified artifacts.

**TABLE 7**
**ANOVA Analysis of the First (N° 1–5) and the Last (N° 4–8) Five Independent Screening Experiments**

| Experimental Series | Statistical Parameters | *p* Values for Growth Rate $r_{(area)}$ | | | *p* Values for Growth Rate $r_{(number)}$ | | |
|---|---|---|---|---|---|---|---|
| | | Day 0–2 | Day 2–6 | Day 0–6 | Day 0–2 | Day 2–6 | Day 0–6 |
| Screening | 1: Exp. No. | 0.897 | 0.893 | 0.969 | 0.501 | 0.818 | 0.973 |
| Exp. N° 1–5 | 2: Treatment | 0.276 | 0.506 | 0.350 | 0.161 | 0.748 | 0.181 |
| | 1/2: Interaction | 0.897 | 0.893 | 0.969 | 0.501 | 0.818 | 0.973 |
| Screening | 1: Exp. No. | 0.364 | 0.669 | 0.596 | 0.203 | 0.761 | 0.861 |
| Exp. N° 4–8 | 2: Treatment | 0.784 | 0.062 | 0.259 | 0.123 | 0.976 | 0.242 |
| | 1/2: Interaction | 0.364 | 0.669 | 0.596 | 0.203 | 0.761 | 0.861 |

*Note:* Independent parameters were experiment number (n = 5, independent experiments) and treatment (n = 2, potencies vs. controls). Data for the nine potency levels (17x, 18x, 21x–24x, 28x, 30x, 33x) and the nine control samples (four samples unsuccussed water, five samples succussed water) were pooled. Measurement parameters were frond area– and frond number–related growth rates for different time intervals (days 0–2, 2–6, 0–6). Data were normalized to the mean of the pooled water controls for every individual experiment.

### Additional Discussion

Growth rate of arsenic-impaired duckweed was increased after application of potentized Arsenicum album regarding both frond area ($p < 0.001$) and frond number ($p < 0.001$) for days 2–6, and by application of potentized nosode (frond area growth rate only, $p < 0.01$). Potencies of gibberellic acid did not influence duckweed growth rate. Due to the inherent use of systematic negative control experiments that did not yield any significant effects and due to various other control calculations, false-positive results can be excluded with very high certainty.

To the best of our knowledge, no study with impaired plants has been published so far that integrated a series of five independent experiments for each potentized test substance as well as five full systematic negative control experiments that had an outcome with comparable significance levels in the very low range[29]. This study is the first that effectually applied a homeopathic nosode preparation to abiotically stressed plants.

In this study, we observed considerable evidence for specific effects of highly diluted homeopathic remedies: Effects of potentized Arsenicum album were clearly different from the zero effects of gibberellic acid, while nosode potencies showed intermediate effects. We thus conclude that the homeopathic potentization procedure (effectuated by serial dilution and succussion) seems to be a specific pharmaceutical process that transmits some genuine properties of the substance potentized to higher dilution levels. Since we used the multiple glass method for preparation of the homeopathic dilutions, material cross-contamination can be excluded. According to our data, the potentization procedure applied seems to exhibit two peculiar characteristics: (1) a nonlinear relationship between successive potentization levels and effect, and (2) specific effects at dilution levels where the probability is extremely low to find any molecules of the diluted substance.

Within the successive series of potency levels 21x–24x of Arsenicum album, 21x–23x stimulated duckweed growth rate, while 24x did not. There seem to be “active” and “inactive” potency levels, a fact reported in almost every investigation that examined series of potencies[12]. Furthermore, Arsenicum album 33x, corresponding to a nominal concentration of $10^{-29}$ g $As_2O_3$/l well beyond the Avogadro limit, also stimulated duckweed growth rate. Similar findings were reported by several other well-controlled studies[28,30,31,32,33,34]. The seemingly irregular groupings of active and inactive potency levels, as well as the non- or ultramolecular effects of very high dilutions, are not only in clear discordance with a classical molecular-based sigmoid dose-response relationship[20], but are also in clear discordance with hormetic effect models[35]. The phenomena observed are not suggestive of molecular-based interactions of material nature, but might occur in the context of force-like (immaterial) resonance effects. However, the nature of any such effect is still elusive. Further research towards the mode of action of homeopathic preparations is important.

Homeopathic Arsenicum album and nosode preparations led to an increase in growth rate of the arsenic-impaired duckweed. This might be interpreted as a decontamination or system recovery effect. Future research must reveal the specific nature of the biological effects induced in duckweed. Compared to other studies with impaired organisms, the measured effect induced by homeopathic preparations in the arsenic-impaired duckweed is rather small (Arsenicum album, growth rate $r_{(area)}$ for days 2–6: +1.2% compared to the water controls)[36,37]. This might be partly due to the very high stability (Table 1) and reproducibility (Table 2) of the test system, the enhancement of which was our primary goal. Correspondingly, the effect size is of medium magnitude ($d = 0.39$).

The treatment of *healthy* duckweed with potentized gibberellic acid induced a significant decrease in growth rate, even for single potency levels[19]. In our experiments with arsenic-impaired duckweed, the application of potentized gibberellic acid did not result in any significant effect, neither increase nor decrease. Since the coefficients of variation of both duckweed bioassays were similar, we assume that the impaired condition of the organisms was responsible for the lacking effect. It seems that gibberellic acid is not the right homeopathic remedy for arsenic-impaired duckweed. In case that the decreasing effect of potentized gibberellic acid onto healthy duckweed could be interpreted as a homeopathic drug proving, the arsenic-impaired organisms were possibly too severely weakened to be able to react to potentized gibberellic acid.

We did not observe any effect of the succussion procedure itself in this bioassay. Interestingly, significant effects of potentized water (compared to unsuccussed water) have been observed in other studies[31,32]. The *potentized* water samples used in the latter investigations differ from the *successed* water samples used in our study by the fact that successed water is succussed only once, and not further serially diluted, thus corresponding to water 1x. Potentized water samples were produced by a process of iterative succussion and dilution, and applied in high potency levels (e.g., 30x, 45x). It thus would be interesting to compare succussed (1x) and potentized (e.g., 30x) water samples within the same study with the same bioassay in order to determine whether the effects of potentized water are due to a specific effect of the potentization procedure or due to a difference in system response towards the physicochemical changes induced by the succussion of water in glass vessels (increased level of glass ions, air suspension, and dissolution, etc.)[38,39]. These results are in line with other recent investigations with various biological test systems where no significant effects of water succussion have been observed[19,40,41]. In further studies, one may compare changes in element concentrations with bioassay responses for different hydrolytic glass qualities.

Potentized remedies may cause an equilibrating effect on variance[42]. In order to test this assumption, all single experiments with Arsenicum album (growth rate $r_{(area)}$ days 2–6) were analyzed by a Levene's test for a difference in variance between the pooled potency levels and pooled controls. No significant result was found. Mean values of coefficient of variation of growth rate $r_{(area)}$ days 2–6 for all experiments with Arsenicum album were 3.10% (potency levels 17x–33x) and 3.11% (controls c0, c1). Assuming that potentized remedies may induce an equilibrating effect on variance, the question is open whether this effect must be imperatively decreasing. Possibly an extremely small variance in a highly standardized bioassay may be increased to a larger variance as usual in natural systems. Thus, the results of this study (with a very small variance) do not argue against the hypothesis of an equilibrating effect of homeopathic remedies on variance.

### Outlook

For future use, the present experimental setup might be optimized by tuning several experimental parameters, e.g., modalities of application, time of impairment in relation to time of homeopathic treatment, measurement time, and growth conditions (light and temperature regime). Another way to enhance the effect size of the test system could be to restrict the range of the tested potency levels to the "active" potency levels and to correspondingly increase the number of replicates per potency level. A particularly interesting range might be 18x–23x since pronounced effects of these four potency levels have been observed in this study. Additional precautions for cross-over contamination should be introduced (e.g., less motion during measurement, additional shielding between experimental conditions). Furthermore, it will be interesting to test a combination of remedies (Arsenicum album and nosode). Specific experimental setups will have to be designed to answer the question of which way homeopathic remedies may influence the variance of outcome measures.

Future applications of this test system can be seen in testing the influence of certain pharmaceutical procedures (e.g., autoclavation, trituration vs. dilution, machine potentization) or other external influences (e.g., heat, light, electromagnetic radiation) that might affect stability and quality of homeopathic preparations. The mode of action is also a possible object of investigation.

### CONCLUSION

The present experimental setup with arsenic-impaired *L. gibba* is a suitable tool to investigate detoxifying effects of potentized substances. Application of potentized Arsenicum album yielded significant effects compared to water controls for the outcome parameters frond area and frond number ($p < 0.001$, $F$-test). The small coefficient of variation (≈1%) and the possibility of pooling individual potency levels (due to the equilibrating character of every single potency level) were the key features of this sensitive and simultaneously stable test system.

### ACKNOWLEDGMENTS

The authors thank Divya Pathak, Tanja Mendonça, and Roland Gosteli for laboratory assistance, as well as Silvia Ivemeyer, Dr. Ursula Wolf, and Amyn Bugaighis for helpful comments. This investigation was funded by Weleda AG (Arlesheim, Switzerland). The sponsor had no influence whatsoever upon design, conduct, and evaluation of the investigation; the decision to publish; and the contents of the manuscript. Additional material support of the Karl und Veronica Carstens-Stiftung (Essen, Germany) is gratefully acknowledged.
